# Osmotic Demyelination: From an Oligodendrocyte to an Astrocyte Perspective

**DOI:** 10.3390/ijms20051124

**Published:** 2019-03-05

**Authors:** Charles Nicaise, Catherine Marneffe, Joanna Bouchat, Jacques Gilloteaux

**Affiliations:** 1 URPhyM—NARILIS, Université de Namur, 5000 Namur, Belgium; joanna.bouchat@unamur.be (J.B.); jacques.gilloteaux@unamur.be (J.G.); 2 Laboratory of Glia Biology (VIB-KU Leuven Center for Brain & Disease Research), Department of Neuroscience, KU Leuven, 3000 Leuven, Belgium; catherine.marneffe@kuleuven.vib.be; 3 Department of Anatomical Sciences, St George’s University School of Medicine, Newcastle upon Tyne NE1 8ST, UK; jgilloteaux@sgu.edu

**Keywords:** astrocytes, oligodendrocytes, osmotic demyelination syndrome, myelinolysis, myelin loss

## Abstract

Osmotic demyelination syndrome (ODS) is a disorder of the central myelin that is often associated with a precipitous rise of serum sodium. Remarkably, while the myelin and oligodendrocytes of specific brain areas degenerate during the disease, neighboring neurons and axons appear unspoiled, and neuroinflammation appears only once demyelination is well established. In addition to blood‒brain barrier breakdown and microglia activation, astrocyte death is among one of the earliest events during ODS pathology. This review will focus on various aspects of biochemical, molecular and cellular aspects of oligodendrocyte and astrocyte changes in ODS-susceptible brain regions, with an emphasis on the crosstalk between those two glial cells. Emerging evidence pointing to the initiating role of astrocytes in region-specific degeneration are discussed.

## 1. Introduction

Osmotic demyelination syndrome encompasses a broad symptomatology from disorientation, slight confusion, paresis and memory loss to seizure, unresponsiveness and coma, depending on the degree of myelin loss in the pons and the presence of extrapontine lesions [1,2,3]. This clinical picture is often linked with an abrupt variation of plasma osmolality, although a variety of cases have now been reported independently of any electrolyte disturbances (alcoholism, malnutrition, cirrhosis, liver transplantation, AIDS, folate deficiency, hyperglycemic states) [4]. Most of the cases are iatrogenic and occur when, in a context of a chronic (>48 h) and profound (<120 mEq/L) hyponatremia, low serum sodium levels are quickly corrected using an abrupt NaCl gradient in an attempt to reach normal ranges of natremia. In the first autopsy reports described in 1959, demyelinated areas were identified in the centro-pontine region as symmetric, sharply outlined lesions confined to the center of the median raphe, and hence led to the seminal appellation of “central pontine myelinolysis” [5]. The lesion seemed to spread out from the midline area of the basis pontis with relative sparing of the adjacent corticospinal and corticobulbar tracts. Extensions to the tegmentum and mesencephalon were observed in some of the largest lesions. In a retrospective and systematic examination of 58 cases, central pontine myelinolysis was combined with extra-pontine involvement in 31% of the cases [6]. The cerebellum, lateral geniculate body, thalami, basal ganglia, subcortical white matter and midbrain structures are the most frequently affected extra-pontine regions and, from a cytoarchitectonic point-of-view, contain a rich apposition of gray and white matter [7]. Singularly, the highly myelinated fibers such as those of the corpus callosum or the anterior commissure are spared from any demyelination. Macroscopic analysis of first autopsy cases identified triangular, T-, bat- or diamond-shaped areas of discoloration in the basis pontis. Microscopic analysis revealed in the pre-identified lesions striking myelin abnormalities including swollen myelin sheaths, myelin fragmentation and demyelinated fiber bundles with concomitant loss of oligodendrocytes, and, if these cells were still found, they were shrunken, damaged or even necrotic [5,8]. Nerve cells and axis cylinders within the lesion were relatively well preserved. As human samples of pons were often analyzed at advanced stages of the disease, neuroinflammation was prominent, in the form of activated microglia and myelin debris scavenged by these macrophages. Peri-lesional astrocytes were hypertrophied and, in old lesions, there could be a central area of cavitation. Due to the highly specific and reproducible location of the demyelinating lesions, the authors who described the seminal case series named the disease “central pontine myelinolysis.” At that time, the term “demyelination” was avoided in order to distinguish this condition, wherein myelin loss appears independently of inflammation, from the multifocal perivascular lymphocytic infiltrates found in multiple sclerosis. It was only later that “osmotic demyelination syndrome” was adopted, gathering both centropontine and extrapontine manifestations in a same pathological entity.

## 2. First Proposed Etiologic Mechanisms

As the centropontine lesions were symmetrical and constant in location, with a sudden onset of symptoms, the syndrome was first proposed as having a toxic or metabolic etiology. In his report, Adams and colleagues concluded in favor of an exogenous or endogenous intoxication, or some deficiency of essential substance to the metabolism of nervous cells [5]. Autopsy examination in the pons of CPM cases revealed large areas of yellowish or greenish discoloration, suggesting red blood cells and hemoglobin degradation subsequent to blood‒brain barrier breakdown. Damage to myelin sheaths and oligodendrocytes was therefore explained by exposure of nerve tissue to unidentified blood-borne component(s) endowed with myelinolytic properties. Rapidly, the question arose as to why some CNS regions were more vulnerable to demyelination than others. The anatomical architecture of the basis pons is singular in that gray matter and white matter are intermixed in such a way that white matter-embedded oligodendrocytes are in close proximity to capillary rich gray matter, making them more exposed to osmotic variation and extravasated blood components. While blood‒brain barrier (BBB) disruption with extravasated red blood cells and blood proteins has been demonstrated in ODS patients and experimental models, the link to oligodendrocyte loss and the time it occurs are still elusive. Intriguingly, the neuron cell bodies and neurites are spared as well as the endothelial cells showing undisturbed tight junctions and barely mild swollen intracellular organelles. Although the mechanism of injury is not clearly defined, both clinical and animal studies support the central role of osmotic stress, most commonly due to a rapid correction of chronic hyponatremia. For a long time, the role of sodium has been underappreciated because in the early years of internal medicine (1960–1970), serum electrolytes were not routinely measured. In the seminal case of Adams et al., hypokaliemia was suspected because of the electrocardiogram abnormalities [5]. Nevertheless, the correction of the confirmed hypokalemia did not modify the neurological manifestations of the patient.

## 3. The Electrolyte Disturbance Hypothesis and Lessons from Experimental Models

Although Adams and others described the first cases of CPM in humans without identifying the cause of demyelination, an earlier report released in 1923 by Rowntree described cases of water intoxication in four dogs that received hypertonic fluid therapy and (two of them) developed neurological manifestations [9]. Symptoms were, unfortunately, not correlated with any brain morphological changes. The first suggestion of electrolyte disturbances and rate of correction of plasma sodium as etiological factors is usually credited to Finlayson in 1973 or Tomlinson in 1976 [10,11], even though Adams mentioned earlier in a conference abstract dehydration and electrolyte imbalance in two cases of non-alcoholic CPM [12]. In 1982, Norenberg, who performed the autopsy of 12 ODS human cases with proven demyelinating lesions, underscored a strong link between the occurrence of ODS and the correction of a hyponatremic condition [13]. By reviewing the medical history, he found the natremia of all patients was raised by more than 20 mmol/L within three days. He also noted that hyponatremic patients who were corrected using gradients less than 20 mmol/L or for a longer time period did not develop symptoms of CPM. Several other studies supported the association between chronic hyponatremia, its overcorrection and CPM [14,15]. Data from experimental studies performed on dogs, rats and rabbits strengthened the fact that the rate of sodium correction after a hyponatremia is one of the most correlated etiological factors [16,17,18]. Remarkably, the demyelinating lesions in dogs appear in the exact same brain areas as reported in human cases, while the central pons is usually spared in rats [19]. Despite these convincing studies in rats, dogs and rabbits, controversy has persisted because a group of patients has remained that develops CPM without having undergone hyponatremia or significant elevations in serum sodium, suggesting the involvement of a third variable.

## 4. Current Understanding of ODS Pathogenesis

### 4.1. Features of the Brain

Unlike other organs, the brain is tightly enclosed in the skull; this physical constraint necessitates specific mechanisms of adaptation to prevent deleterious volume organ expansion. Volume changes are particularly harmful to neurons, whose function is vulnerable to excessive cell swelling or shrinkage, and millions of neural networks made of long and targeted neurites, whose function relies on the integrity of their connectome. Volume alterations can greatly impact excitability, metabolism and induce apoptosis in nervous cells. Therefore, the brain has developed osmo-regulatory mechanisms allowing it to cope with a physiological range of osmotic fluctuations. In other words, changes in extracellular osmolality will systematically lead to adaptation of brain cell osmolality. Change in intracellular osmolality is an adaptive function and results from either an increase or a loss of intracellular osmolytes. According to the paradigm that ODS develops into a slowly established and severe hyponatremia that is too rapidly corrected, the following paragraphs decipher how the pathophysiological mechanisms are currently understood.

### 4.2. Response to Hyponatremia

When hyponatremia settles, the brain cell intracellular compartment becomes progressively hypertonic compared to the interstitial fluid. In theory, in the absence of any adaptation, extracellular water would move freely across membranes from an area of low solute content into cells with a high solute content, leading to cellular swelling. In the brain, glial cells have a particular role of water handling and buffer the water movements in and out of the CNS. For instance, during hypotonic stress due to water overload, glial cells selectively swell whereas neurons do not. Astrocytes express at high levels of water permeable channels, AQP1 and AQP4, and are in close contact with the blood‒brain barrier, a crucial position for regulating influx and efflux of water or ions. The role of glial AQP4 in brain water homeostasis has been characterized by the team of Verkman, who showed that AQP4^−/−^ mice had improved survival and less cerebral edema than wild-type mice in a model of brain edema caused by acute water intoxication [20,21].

To counteract excessive cell swelling, glial cells lose inorganic osmolytes (first Na^+^ and Cl^−^, then K^+^) over a period from few minutes to 24 h, which sustains an AQP4-mediated water efflux. This adaptation is an energy-dependent phenomenon that requires the activation of Na‒K‒ATPases that first expel Na^+^, followed by the extrusion of K^+^ and Cl^−^ through K^+^ channels, the K^+^‒Cl^−^ co-transporter and the volume-sensitive Cl^−^ channel [22,23,24,25,26]. Brain edema would occur if the osmolyte depletion rate is overridden, like during acute water intoxication.

It has been demonstrated that the maximum loss of intracellular cations (Na^+^ and K^+^) that occurs during hyponatremic conditions is 18% compared to iso-osmotic conditions; hence, it has been theoretically calculated that the corresponding natremia would be equal to 103 mmol/L. However, hyponatremia below 100 mmol/L is not infrequent in human practice and not incompatible with long-term survival, as evidenced in rodents, likely suggesting additional mechanisms of adaptation [27]. It was demonstrated that cells exposed to sustained hyponatremia were also losing organic osmolytes over time [28]. This includes the extrusion of myoinisotol, betaine, creatine, taurine, glycine, aspartate, glutamine and glutamate, with glutamine and taurine being the most severely depleted at the end of such adaptative processes [29]. Interestingly, the efflux is delayed compared to inorganic osmolytes (started over a day and completed within 48 h) [30]. Hence, its completion defines the empirical threshold of acute (<48 h) versus chronic (>48 h) hyponatremia. Overall, the depletion of inorganic and organic solutes drives the process of regulatory volume decrease and establishes an “adapted” osmotic balance (shifted to hypotonicity) between the brain interstitium and the intracellular compartment.

As hyponatremia is a medical condition associated with significant morbidity (osteoporosis, gait instability, fractures, deficits of attention) and increased mortality, EU and U.S. panel experts have issued recommendations to properly manage the hyponatremic condition [31,32].

### 4.3. Following Correction of Hyponatremia

Things get complicated when the tenuous and previously reached osmotic equilibrium is challenged by an abrupt correction of hyponatremia. If the correction occurs too rapidly, typically using a bolus of hypertonic saline, osmolyte-depleted cells have to adapt towards a hypertonic interstitial fluid, unless undergoing rapid cell shrinkage. The repumping of inorganic electrolytes from the interstitial fluid can take place within minutes by activating ATP-dependent membrane ion pumps, in the hope of restoring the intracellular inorganic ion content and osmotically driving water influx. Although the rapid uptake of inorganic electrolytes helps to restore the size of shrunken cells, it elevates the intracellular ionic concentration. Because high ionic strength can disturb the intracellular folding of proteins or protein‒protein associations, this condition is not sustainable in the long term and cells must progressively organize a secondary response, based on the accumulation of organic osmolytes. Once inorganic ion shifts have been exhausted, if the rise of tonicity is faster than the rate at which organic osmolytes can be transported or de novo synthesized, the cell will ineluctably shrink. Re-accumulation of intracellular organic osmolytes occurs at a slower rate and represents a significant metabolic burden [30,33,34]. If, during the process of intracellular osmolyte recovery, there is an imbalance between intracellular and extracellular osmolality, glial cells that sheath the synaptic compartments or axonal processes will be subject to cell shrinkage, which may lead to irreversible cellular damage, induction of apoptotic cascade and disruption of neuropil integrity [35,36]. In a few cases of human ODS lesions resulting from solely hypernatremia, ODS is proposed to occur as a result of hypertonic insult, in which the interstitial fluid becomes hypertonic faster than the rate at which the brain cells can compensate by synthesizing or taking up osmolytes [2,37,38,39].

It remains unclear why there is such a regional patterning in CPM and EPM lesions, with the pons, basal ganglia, mesencephalon or deep cortical layers more vulnerable than areas with higher myelin content. In 1995, Lien and colleagues nicely correlated the fact that ODS-susceptible regions were those that had a significant delayed recovery of organic osmolytes (glutamine, taurine, myoinositol, and creatine), and subsequently those that will fail to optimally manage rapid changes in osmolality [40]. To date, the cell type accounting for the largest accumulation or depletion of those osmolytes is unidentified. Furthermore, the fact that exogenous myoinositol administration improves survival and reduces myelin loss in a rat model of ODS is a very strong indicator of the role of organic osmolytes [41].

### 4.4. Blood‒Brain Barrier Disruption

The concept of BBB disruption is tempting but still debated. Brain endothelial cells are the first cells that must face the rapid variations of plasma osmolality and whose barrier functions could be compromised during regulatory volume processes. This hypothesis arose from autopsy observations showing areas of greenish and yellowish discoloration in the basis pontis of CPM patients, suggesting erythrocyte’s extravasation with subsequent hemoglobin degradation. Disruption of the BBB has been seen in experimental models of ODS [42,43,44]. The initiation of the opening, the degree, the duration of disruption and its contribution to disease progression are matters of speculation. Interestingly, the observation of the BBB opening is regionally correlated to brain areas that are or will be undergoing myelin and oligodendrocyte loss. Rojiani et al. demonstrated leakage of peroxidase from intravascular compartment and slightly increased brain water content as soon as 3 h post-correction of chronic hyponatremia in rats [45]. This would suggest that the BBB is compromised early. Ultrastructural analysis of endothelial cells evidenced high pinocytotic activity and focal inter-endothelial fluid accumulation [46]. Perivascular astrocytic foot processes and neurites were markedly swollen. All these morphological alterations tended to normalize at 48 h post-correction. Using immunohistochemistry, Baker et al. showed extravasation of endogenous IgG and the C3d split-fragment of activated complement (150 and 187 kDa, respectively) at 20 h and five days after rapid correction of hyponatremia in rats, which further strengthens that large blood-borne molecules invade the brain parenchyma [42,43]. Once endothelial cell junctions become permeable, small and large plasma molecules such as serum complement, immunoglobulins, cytokines, and other inflammatory mediators (e.g., nitric oxide released by damaged endothelial cells, etc.) have facilitated access to brain parenchyma and would damage oligodendrocytes [47]. Using albumin extravasation and IgG, Bouchat et al. showed that BBB breakdown was delayed compared to oligodendrocyte and astrocyte damage in a mouse model of ODS [44]. To our best knowledge, all markers of BBB impairment that were ever used in experimental works have a molecular weight equal or above 44 kDa (horseradish peroxidase) [45]. Those tracers or plasma proteins extravasate in the case of significant BBB damage, which does not mean that minimal alterations of the barrier might already occur during chronic hyponatremia or immediately after the correction of hyponatremia; this should be investigated using smaller intravascular tracers. Regardless of their size, blood protein extravasation favors water entry through disrupted endothelial cell junctions and contributes to local vasogenic edema [46]. Remarkably, no inflammatory cells (polymorphonuclear or lymphocytes) cross the BBB during the ODS course [48]. Although hemoglobin and degradation by-products were found in late ODS lesions of human autopsy cases [5], red blood cells and hemosiderin deposits were never seen in the brain parenchyma of ODS animal models [42,43,44]. It has been demonstrated that hemoglobin or other blood-borne molecules are toxic to neurons and oligodendrocytes [49,50,51,52,53,54]. If BBB leaks, which more than likely are non-selective, play a significant role in ODS pathology, the difficulty resides in explaining the selective loss of oligodendrocytes with the relative sparing of neurons in the same demyelinated areas. An increase in the permeability of the BBB has been well documented in other models of neurological disorders; however, such a specific pattern of demyelination does not occur. These facts strongly imply that the opening of BBB could count as an epiphenomenon, secondary to damage at the peri-vascular astrocyte end-feet or to neuroinflammation, which is distinct from the primary osmotic insult. Finally, glucocorticoid therapy mitigates the neurological and histopathological outcomes in experimental paradigms [55,56,57,58,59]. It has been postulated that the effect observed was due to the prevention of the BBB opening and/or restoration of its integrity.

## 5. From a Myelinating Oligodendrocyte Pathology

Mature oligodendrocytes arise from oligodendrocyte progenitors and neural stem cells and are the cells dedicated to axon myelination in the CNS [60]. Myelin sheaths are a cellular expansion from the oligodendrocytes, made of a vast number of membrane lipids and, to a lesser extent, of integral proteins involved in the coherence of the multi-layered ensheathment [61]. The CNS myelin proteins are proteolipid protein (PLP), myelin basic protein (MBP) and glycoproteins (such as myelin associated glycoprotein (MAG)), and myelin oligodendrocyte protein (MOG)). PLP is the most abundant protein in the central myelin. The highly lipid-rich myelin membrane acts primarily as an insulator for axons, allowing high-velocity and energy-sparing electrical conduction. In mammals, myelination is largely a postnatal event relevant to oligodendrocytes. After the completion of myelin biogenesis, oligodendrocytes are still committed to preserving long-term myelin stability and functionality. Diseases affecting myelin development (hypomyelination or dysmyelination) or characterized by acute or chronic myelin destruction (demyelination) lead to profound alterations of electrical conduction. Neurological symptoms will be related to the anatomic site of demyelination. The term demyelination, such as used in ODS, describes a loss of myelin with a relative preservation of axons [2]. Demyelination results from disease affecting the myelin sheaths themselves or the oligodendrocytes that form them. Although an axon that has been chronically or severely demyelinated tends to atrophy and degenerate, demyelinating diseases exclude those in which axonal degeneration occurs first and degradation of myelin is secondary (e.g., Wallerian degeneration).

During ODS, both myelin and oligodendrocytes degenerate. All the anatomopathological features were fully described by the original authors upon microscopic observation of the first cases of CPM [5,12]. Lesions included a range of histological alterations from mild intramyelinic and intracellular edema to prominent swelling of the myelin sheaths, obvious myelinolysis with oligodendrocyte degeneration; axon and neuronal cell bodies were preserved except at the very epicenter of the lesions; lymphocytic infiltrates were absent, although myelin-loaded macrophages and microglial cells were observed; no hallmark of liquefactive necrosis was observed. Subsequent reports indicate that many ODS lesions are not demyelinating stricto sensu, given the concomitance of axonal degeneration and myelin loss, especially in the epicenter of severe and long-established lesions [62,63,64]. Because of this singular pathological picture, the clinical identity of ODS is distinguishable from brain hypoxia and from primary inflammatory demyelinating diseases [65]. In experimental models, cell markers of oligodendrocytes such as CNPase (2′,3′-cyclic nucleotide-3′-phosphodiesterase), APC (Adenomatous Polyposis Coli), O4 or Olig family proteins are used in immunohistochemistry to evidence the cell-specific loss in demyelinating-prone areas [44,48,66]. In addition, staining (e.g., luxol fast blue, eriochrome R cyanin) and protein markers such as MBP, PLP, MOG, MAG are used to characterize the demyelinating nature of the lesions, keeping in mind that histological stains showed less specificity towards myelin subcomponents and that myelin proteins may be more or less susceptible to rapid degradation following myelin attack [67,68].

Oligodendrocytes are privileged targets in a number of neurological diseases; a striking example is hypoxic brain damage [69,70]. Apoptosis is often mentioned as the preferred mechanism of cell death when it comes to oligodendrocyte death. Caspase-dependent activation of apoptosis in oligodendrocytes results in rapid focal demyelination—within 24 h of cell loss [71]. Whether myelin or oligodendrocyte is the primary target during ODS is still elusive, but there is some evidence for a role of apoptosis in ODS. Using immunohistochemistry on human autopsy material, De Luca found an imbalance between pro- and anti-apoptotic markers, with a shift toward increased expression of apoptotic-related markers, death receptor 3, Bax, and Bak in glial cells [72]. Cellular stresses contributing to oligodendroglial death are numerous: oxidative stress, excitotoxic insults, or volume changes [69,73,74,75,76]. It is well established that the persistent physical shrinkage induced by hypertonic stress leads to cell death in a variety of cell types. For example, it has been shown in thymocytes lacking an efficient regulatory volume increase (RVI) response that hypertonic conditions result in sustained cell shrinkage, leading to the activation of the apoptotic process, whereas other cell types that have an RVI response were resistant to hypertonic-induced cell death [77]. In that respect, rat brains exposed to systemic hypertonic conditions following mannitol infusion do not undergo massive cell death but elicit an osmo-adaptative response, mostly in neurons and oligodendrocytes, as attested by tonicity-induced expression of transcription factor TonEBP and increased expression of SNAT2, a neutral amino acid transporter involved in their accumulation during volume recovery of shrunken cells [78]. It is important to note that the magnitude of hypertonic challenge in this experimental setting was to a large extent greater than the amplitude of correction of hyponatremia imposed in a rat ODS model, meaning that hypertonic challenge per se is insufficient to induce oligodendrocyte death. Alternatively, the authors of the study have hypothesized that chronic hyponatremia would lead to the downregulation of signaling pathway regulating SNAT2 expression. As a result, during the rapid correction of hyponatremia, no or delayed tonicity-induced expression of SNAT2 occurs in oligodendrocytes. Hence, the accumulation of amino acids is impaired and cannot counteract the elevation of extracellular tonicity or intracellular ionic strength. Since oligodendrocytes probably rely primarily on amino acid accumulation to recover their cell volume, they remain shrunken and trigger activation of apoptotic pathways. Hypothetically, the other brain cells that do not rely on SNAT2-mediated amino acid accumulation for osmo-adaptation are less affected.

In addition, oligodendrocyte death can be elicited by exposure to inflammatory cytokines during acute or chronic neuroinflammation. In mice, local production of TNF- α by either astrocytes or microglial cells potently and selectively induces p55TNF receptor-mediated oligodendrocyte apoptosis and myelin vacuolation, even in a context of intact blood‒brain barrier and the absence of immune cell infiltration [79]. Numerous lines of evidence demonstrate the secretion of pro-inflammatory mediators (TNF-α, IL-1α, IL-1β IL-6, iNOs) by astrocytes and microglia in experimental ODS models [66,80]. In such models, therapeutic interventions aiming at microglia inhibition [80,81,82,83] or antibody-based TNF-α neutralization (Infliximab) [66] led to significant improvements in neurological signs and less severe demyelination. If not the primary event in ODS, secondary inflammation with microglia activation might exacerbate the oligodendrocyte injury inside the demyelinating lesions.

Intercellular connections between astrocytes and oligodendrocytes are vital for the proper functioning of oligodendrocytes [84]. Gap junctions support the metabolic and electric coupling of glial cells, affording the exchange of small molecules (<1.5kDa) such as water, ions (Ca^2+^), ATP or glio-/neurotransmitters from one cell to another. Evidence shows that astrocyte gap junctions and astrocyte proximity are essential for oligodendrocyte myelination activity [85,86]. This is illustrated by the lethal phenotype of Cx47/Cx30 or Cx32/Cx43 dKO mice in which astrocyte‒oligodendrocyte communication is disturbed [87]. Interestingly, Cx47/Cx32 dKO mice, ablated for major oligodendrocyte connexins, are not lethal but show abnormal myelin development, oligodendrocyte apoptosis and axonal degeneration [88,89]. Finally, Cx30/Cx43 dKO mice (ablation of major astrocyte connexins) develop white matter pathology with vacuolated oligodendrocytes and intramyelinic edema [90,91]. During experimental ODS, we and others have demonstrated an early loss of both astrocyte and oligodendrocyte connexins, especially in ODS-susceptible brain regions [44,48]. For instance, Cx43 and Cx47 expression, as well as Cx30, is downregulated in the thalamus of an ODS mice model. Dysregulation of major astrocyte‒oligodendrocyte connexins could therefore participate in the local demyelination process, and seems to concur with what has been observed in knockout mice.

The regional susceptibility of oligodendrocytes in ODS remains elusive. Most regions affected by demyelination in ODS are made of grey‒white matter juxtapositions, rather than pure white matter tracts. It has been known for decades that oligodendrocytes populating grey matter are morphologically different than those found in large fiber tracts. Originally, oligodendrocytes have been classified based on morphological criteria [92,93,94,95,96,97,98,99]. Type I and II oligodendrocytes populate the grey matter and fine white matter tracts, where they myelinate small caliber axons with short internode length. Type III and IV oligodendrocytes myelinate large-diameter axons with longer internodes, forming myelin sheaths whose thickness can reach 50 times that of type I/II oligodendrocytes [96,98,100]. The phenotype of oligodendrocytes and the features of the myelin they build are largely dependent on the axons to which they associate [101]. Adding to this structural heterogeneity is the recent description, in the mouse brain, of 13 distinct Pdgfrα(+) oligodendrocyte populations based on their differential transcriptional profiles [102].

## 6. Towards an Astrocyte Pathology

**Figure 1:**
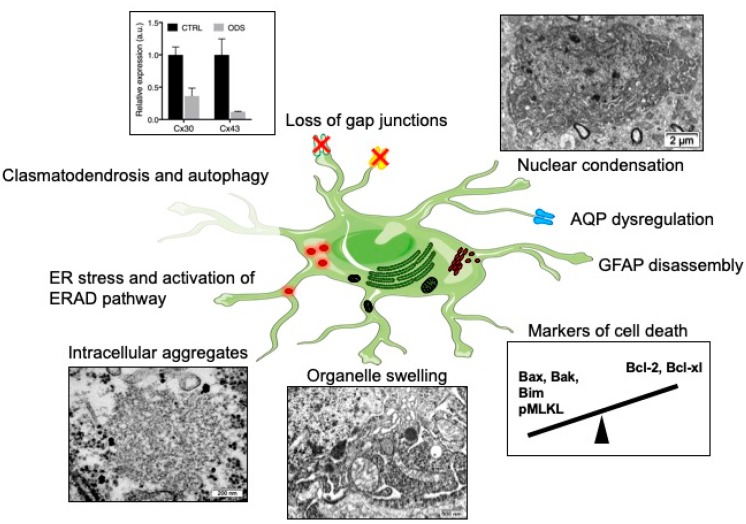
Astrocytopathy during osmotic demyelination. Major changes observed in astrocytes of demyelinating-prone regions include: downregulation of gap junctions (Cx30 and Cx43) [44,48], dysregulation of aquaporin expression (AQP1 and AQP4) [113], ER stress and activation of ER-associated degradation (ERAD) pathway [112], intracytoplasmic aggregates [112,114], swelling of various organelles [44,46,114], loss of GFAP intermediate filaments [6,48], clasmatodendrosis [114], nuclear condensation and upregulation of cell death markers [44,48,72,112].

Astrocytes are indispensable for myelination; they regulate several aspects, from the trophic support of oligodendrocytes to the myelinating activity of oligodendrocytes, and aid in removal of myelin debris [85,103,104,105,106,107,108,109,110]. Several lines of evidence show that astrocytes are a prime susceptible cell type during ODS. Astrocyte-specific pathological changes are summarized in Figure 1. The first clues pointed to decreased expression of GFAP in the lesions of ODS rat model and in old demyelinated lesions of human cases (Figure 1) [6]. This change in glial cell marker expression could be related to in vitro results already describing GFAP downregulation after cultured astrocytes had been exposed to a cocktail of pro-inflammatory cytokines [111]. Solid evidence of astrocyte death was later produced by Gankam-Kengné et al. in a rat ODS model. Using a modified TUNEL assay and gammaH2AX immunolabeling, the authors observed DNA fragmentation and chromatin condensation, which usually drive the early steps of apoptosis (Figure 1) [48,112]. This cell death was specifically detected prior to myelin protein marker loss (i.e., MBP) and strikingly in most of the astrocytes of the future demyelinated areas [48]. Later, the same authors strengthened the findings that the correction of a chronic hyponatremia disturbs the apoptotic balance in the brain cells, by favoring the expression of pro-apoptotic proteins Bax and Bim, while repressing anti-apoptotic proteins Bcl-XL and Bcl-2 [112]. Studying the temporal relationship between glial cell loss and demyelination in ODS mouse model, Bouchat et al. found that astrocyte cell markers as well as oligodendrocyte cell marker loss also preceded myelin loss [44]. Intriguingly, lesions in mice were not associated with any apoptotic markers but rather with phosphoMLKL-immunoreactivity, a marker of necroptosis.

Recently, Gankam-Kengné et al. showed in their experiments that astrocytes of the demyelinating-prone regions accumulated large amount of insoluble, polyubiquitinated and aggregated proteins (Figure 1) [112]. Singularly, those peculiar accumulations of protein materials were absent from oligodendrocytes of the same brain regions and were seldom observed in neighboring neurons. Concomitantly, the rapid correction of hyponatremia induced the expression of markers of endoplasmic reticulum (ER) stress, the activation of ER-associated degradation pathway and autophagy. It was shown 10 years ago in worms that acute loss of intracellular water following exposure to hypertonic conditions causes a transient increase in the cytoplasmic ionic strength [115,116]. This ultimately leads to improper protein folding, direct protein damage with loss of enzymatic activity, abnormal protein‒protein interaction and even protein aggregation. In this model, if osmotically challenged cells have enough time to adapt, for instance along a slow establishment of hypertonicity, they are able to trigger intracellular pathways supporting clearance of abnormal proteins [115]. Moreover, intracellular organic osmolytes act as molecular chaperones contributing to the stability and the solubility of proteins, while certain osmolytes extrinsically help at protein folding [117]. Considering the osmolyte depletion during the adaptation of hyponatremic conditions, it would not be astonishing if, when faced with a relatively hypertonic environment during the correction of hyponatremia, brain cells would be devoid of defense mechanisms against some protein misfolding [118].

Analyzing a mouse model of ODS at the ultrastructure, our group found evidence of serious astrocyte injury. Astrocytes of demyelination-prone regions showed autolytic activities, with membrane whorls and other complex cytoplasm figures exposing autophagocytosis, while huge whorls of astrocyte end-feet self-excisions confirmed the process of clasmatodendrosis. The fragmentation of astrocyte processes contributes to the loss of intercellular contacts and to the fragilization of the neuropil [114]. The perinuclear area as well as the most distal extensions of astrocytes showed swollen cytoplasm, accompanied by endoplasm damage, mitochondria swelling and poor organelle content [114]. Still in the mouse model, drastic downregulation of astrocytic gap junctions was seen as in the rat model [44,48,81] (Figure 1) and occurred at the same time as the loss of other astrocyte cell markers (AQP4, S100b, Aldh1L1). The restricted Cx43 loss in the thalamus was intriguing: thalamic astrocytes seemed to be particularly sensitive to ODS whereas the striatum was entirely spared. Up to nine different subtypes of astrocytes based on morphological criteria had been identified in the CNS. One could hypothesize that this heterogeneity could explain their inconstant sensitiveness towards similar injurious stress. Beside, ion channels, connexin, or cytoskeletal protein expression also present regional heterogeneity [119,120]. For instance, Griemsmann and co-authors combined analyses of mice with deletion of connexins (Cx26^−/−^, Cx30^−/−^, Cx43^−/−^), gene and protein expression and showed that, in the thalamus, the dominant connexin expressed is Cx30, with many cells lacking any expression of Cx43 [121]. In the hippocampus and neocortex, Cx43 is the predominantly expressed connexin. In conditional KO mice, the deletion of Cx43 decreased the coupling in the hippocampus but not in the thalamus. An analysis of Cx30 expression in the rat brain showed its widespread presence in the brainstem and diencephalon [122]. Scattered cells in the hippocampal formation showed moderate intensity of labeling for Cx30 and in telencephalic regions weak or no expression at all was found. While Cx30 and Cx43 are usually co-localized, Cx30 is absent in the white matter. A study has shown that different classes of astrocytes have clonal identity, suggesting that astrocyte heterogeneity is specified early during CNS development [123]. Another study, however, showed that regionally distinct subsets of astrocytes receive Hh signaling (through the Sonic Hedgehog receptor) and attenuation of Shh signaling in postnatal astrocytes resulted in the upregulation of GFAP and cellular hypertrophy. These findings demonstrate a role for neuron-derived Shh signaling in regulating specific populations of adult astrocytes [120,124]. Hence, regional ODS susceptibility might be explained by local neuronal‒astrocyte crosstalk, differential expression patterns and/or differential response to osmotic insult. In order to explore the astrocyte region-specific vulnerability observed in ODS, Takeda and others isolated cells from the rat cerebral cortex and from the cerebellum, then examined how extracellular sodium variations induce astrocyte death and whether the response differed between the two populations of astrocytes [125]. In their study, they showed that apoptosis could be induced in both cell populations by sustained exposure to high extracellular sodium (306 mM) or by pharmacological voltage-gated Na^+^ channels opening. Intriguingly, their findings showed that astrocytes from the cerebellum are more susceptible to extracellular sodium alterations—mimicking the correction of hyposodic conditions—than cerebral astrocytes. The authors hypothesized that this discrepancy between their in vitro data and what is observed in vivo ODS models might be explained by a switch of cell phenotype when astrocytes are cultured.

During the correction of hyponatremia, glial cells are exposed to acute hyperosomolar stress, leading to an immediate in-to-out water shift, followed by active Na^+^, K^+^, Cl^-^ and amino acid re-uptake, with later idiogenic osmoles synthesis. It is becoming more and more obvious that the intracellular Na^+^ concentration ([Na^+^]_i_) plays a major role in astroglial homeostasis and signaling [126,127,128]. For instance, it has been measured that [Na^+^]_i_ ranges from 10 to 17 mM in cultured astrocytes, which is remarkably high compared to cultured neurons (4 to 9 mM). Astrocytes undergo slight fluctuations of this [Na^+^]_i_ that are involved in the regulation of multiple homeostatic cascades. Changes can elevate [Na^+^]_i_ by 10–20 mM and propagate through the glial syncytium under the form of Na^+^ waves. Astrocyte [Na^+^]_i_ signals are elicited by the Na^+^ influx through ion channels or Na^+^-dependent neurotransmitter transporters. Among those last, astroglial glutamate (EAATs) and gamma-aminobutyric acid transporters (GATs) are of importance, because they are crucial for maintaining a low synaptic neurotransmitter concentration. As a corollary, astrocyte [Na^+^]_i_ influences the uptake of glutamate and GABA at the synapse or from the extracellular spaces and thereby has a direct impact on neuronal excitability. Increases in [Na^+^]_i_ can impede re-uptake or even reverse the transport activity. Astroglial GABA transporters are the most susceptible to [Na^+^]_i_ fluctuations and easily switch from a repumping mode to a reverse mode, with significant GABA release [129,130,131]. Moreover, changes in [Na^+^] can also affect the activity of glutamine synthetase, which further impacts the glutamine‒glutamate cycle [132]. Preliminary data obtained by our lab suggest that lesioned astrocytes isolated from the ODS-susceptible region show a drastic downregulation of glutamate and GABA transporters as well as glutamine synthetase at early time points following the correction of hyponatremia (unpublished data). Impaired abilities to uptake or handle crucial neurotransmitters by astrocytes in the cortex, basal ganglia network or thalamus might partially account for the neurological symptomatology observed during ODS.

Astrocytes express both aquaporin-1 (AQP1) and aquaporin-4 (AQP4), highly permeable water channels involved in brain volume homeostasis. Astroglial AQP4 is mainly expressed in the plasma membrane of the perivascular end-feet, where it allows fast and efficient water shifts between intracellular and extracellular fluids. In an experimental ODS model, astrocytopathy is strengthened by the loss of AQP4 immunoreactivity in susceptible brain regions, well before demyelination and, intriguingly, prior to GFAP downregulation (Figure 1) [81]. Autopsy study of six human brains with CPM revealed that in four of the cases a loss of AQP1 and AQP4 was found within the demyelinating lesions [113]. Unlike the rat ODS model, wherein GFAP is downregulated early in brain lesional astrocytes, GFAP expression was preserved throughout the lesional astrocytes of CPM patients, despite the astrocytes having downregulated AQP1 and AQP4 expression. This argues against the loss of aquaporins’ immunoreactivity being secondary to the death of astrocytes. In the same study, although fascicular oligodendrocytes within the lesions showed morphological features of apoptotic cells, neighboring astrocytes did not show signs of cell death. Once again, this stresses that animal models do not fully mimic human pathology and that, somehow, interspecies or inter-regional astrocytic heterogeneity might explain these discrepancies.

## 7. Concluding Remarks

It is still not possible to predict the outcome and the recovery possibilities for patients undergoing or having undergone ODS. While some patients fully recover and remyelinate, within several months, there are patients who only partially recover neurologic functions, patient who do not recover at all or even die [133,134,135]. Therapeutic interventions are nonexistent because early diagnosis remains a challenge, as well as the detection of brain damage following an inadvertent rapid correction of chronic hyponatremia. Clinical guidelines have been issued regarding the proper management of hyponatremia, but no guidelines clearly define the most appropriate measures to undertake once hyponatremia has been overcorrected. In animals, strategies using sodium relowering or urea infusion decreased mortality rate and improved neurologic score. Currently, several human case studies are showing improved neurological outcomes upon serum sodium relowering or urea administration [136,137,138]. Scarce reports of therapy with intravenous immunoglobulin, plasma exchange and thyrotropin-releasing hormone also suggest good neurological recovery in humans but are difficult to interpret due to the small sample size [139,140]. Experimental animal models paved the way for the current understanding of ODS etiopathogenesis and were useful for designing potentially translatable therapeutic approaches in case of overcorrection of hyponatremia. Administration of minocycline [81,82,83], infliximab [66], dexamethasone [56,58] or lovastatin [80] showed reduced neurological dysfunction correlated with less severe brain demyelination in animal models. Most of these compounds target neuroinflammation or the activated microglia that undoubtedly contribute to the disease by exacerbating myelinolysis and glial cell injury. In this regard, early detection of glial stress during ODS would help with diagnosing brain damage and establishing an appropriate clinical management. As demonstrated in human and animal models of ODS, both astrocytes and oligodendrocytes suffer from osmotic stress, with astrocytes likely being among the first nervous cells impacted. This is not surprising given the forward position of astrocytes and their role in regulating water and ion fluxes at the gliovascular interface. When responding to osmotic stress, glial cells release molecules that can serve as biomarkers for ODS progression. Hence, astrocyte-derived markers such as AQP4 and S100β were assayed in the blood of ODS animal models and their concentration was positively correlated with neurological impairments [48,81]. By using a panel of sensitive biomarkers exploring oligodendrocytes’ and astrocytes’ viability, we should be able to detect the first signs of brain damage, prior to the occurrence of demyelinating lesions.

The mechanisms of myelin loss in cell- or region-specific vulnerability towards osmotic insult remain incompletely understood in ODS. Autopsy cases as well as experimental models suggest that prominent degenerative changes in astrocytes precede oligodendrocytopathy and demyelination. The dysfunction of astrocytes has been underestimated and overlooked, considering that brain astrocytes regulate many homeostatic functions in coordination with those of oligodendrocytes and neurons. It seems that, during ODS, the fate of oligodendrocytes is tightly linked to astrocytes’. Surrounding neurons cope relatively well with the osmotic challenge, though myelinated axons swell and sometimes degenerate in the lesion epicenter. The mechanism by which astrocytes and oligodendrocytes locally trigger cell death upon the correction of hyponatremia remains unknown. If hyponatremia status is a prerequisite for demyelination, one can speculate that chronic hyponatremia downregulates the cell pathways involved in the response to relative hypertonicity. Interestingly, experimental data showed that brain demyelination could be induced by hypernatremia using a large osmotic gradient [141], which suggests that the response to cell shrinkage upon exposure to hyperosmotic stimulus may be the key factor. If we could understand how glial cells from ODS-resistant brain regions adapt to chronic hyponatremia and its correction and why they do not initiate cell death, we would greatly advance the understanding of ODS pathology. The answer might come from transcriptomic or metabolomic analysis at the single-cell level.

Hope also lies in the subgroup of patients that shows complete remyelination of the brain lesions after an episode of ODS—or at least undetectable lesions using current brain imaging methods. Future research should investigate how the endogenous repair abilities of the brain are activated, through recruitment, stimulation and differentiation of glial-/oligodendrocyte-progenitor cells, and what lies beneath the cause(s) of success or failure of remyelination. As demonstrated in other models of demyelinating diseases, resident astrocytes and their reactive counterparts are possible sources of oligodendrocyte precursors and may also significantly contribute to remyelination [142].

## Abbreviations

APC	Adenomatous Polyposis Coli
AQP	Aquaporin
BBB	blood‒brain barrier
C3d	Complement component 3 d
CNPase	2′,3′-cyclic nucleotide-3′-phosphodiesterase
CNS	Central nervous system
CPM	Central pontine myelinolysis
Cx	Connexin
EAAT	Excitatory amino acid transporter
EPM	Extra pontine myelinolysis
ER	Endoplasmic reticulum
GAT	gamma-aminobutyric acid transporter
GFAP	Glial fibrillary acidic protein
IgG	Immunoglobulin G
IL	Interleukin
MAG	myelin-associated glycoprotein
MBP	myelin basic protein
MOG	myelin oligodendrocyte protein
ODS	Osmotic demyelination syndrome
Pdgfra	platelet-derived growth factor receptor alpha
Shh	sonic hedgehog
PLP	proteolipid protein
RBC	Red blood cells
RVI	regulatory volume increase
SNAT	Sodium-dependent neutral amino acid transporter
TNF	Tumor necrosis factor
TonEBP	Tonicity-Responsive Enhancer-Binding Protein
